# A modified sol-gel synthesis protocol for high-quality ZnSnO₃ thin films with enhanced electrical and optical properties for energy and sensor applications

**DOI:** 10.1016/j.mex.2025.103629

**Published:** 2025-09-16

**Authors:** M. Rajesh, B. Rajalingam

**Affiliations:** a Department of Computer Science and Engineering, Aarupadai Veedu Institute of Technology, Vinayaka Mission’s Research Foundation (DU), Chennai, Tamil Nadu, 603104, India; b Department of Computer Science and Engineering, School of Computing, Vel Tech Rangarajan Dr. Sagunthala R&D Institute of Science and Technology, Avadi, Chennai, India

**Keywords:** ZnSnO₃ thin films, Sol-gel synthesis, Optical and electrical properties, Gas sensing performance, Transparent electronics

## Abstract

This paper presents a modified sol-gel synthesis methodology for producing high-quality ZnSnO₃ thin films with improved optical transparency, electrical conductivity, and gas-sensing capabilities, ideal for renewable energy and sensor applications. Adjusting the annealing temperature from 200 to 500 °C allowed for precise control of phase, transparency, and conductivity. X-ray diffraction showed that temperature drives the transformation from amorphous to crystalline phases.

Films annealed at 350 °C exhibited over 85 % transmittance in the visible spectrum and a direct band gap of 3.3 eV, making them excellent candidates for transparent electrodes in future photovoltaic and optoelectronic systems. At 450 °C, electrical testing revealed a low resistivity of 5.2 × 10⁻³ Ω·cm, representing a significant improvement over typical ZnO-based transparent conductive oxide.

Gas-sensing studies showed strong responses—75 % for CO₂ at 250 °C and 70 % for NO₂ at 300 °C—with more than 95 % retention after 50 cycles, indicating long-term stability. Energy-efficient transparent electronics, environmental monitoring, and high-sensitivity gas sensors can be reproducibly and scalably fabricated using the updated sol-gel technique.

• XRD and UV–Vis corroborated the temperature-driven transformation from amorphous to crystalline ZnSnO₃. Films annealed at 350 °C had a 3.3 eV band gap and over 85 % visible transmittance.
• At 450 °C, resistivity decreased to 5.2 × 10⁻³ Ω·cm, resulting in a conductivity of 192 S·cm⁻¹, exceeding standard ZnO TCOs.
• Gas sensitivities were 75 % for CO₂ (250 °C) and 70 % for NO₂ (300 °C), with 95 % stability retained after 50 cycles.

## Graphical abstract

**Figure:**
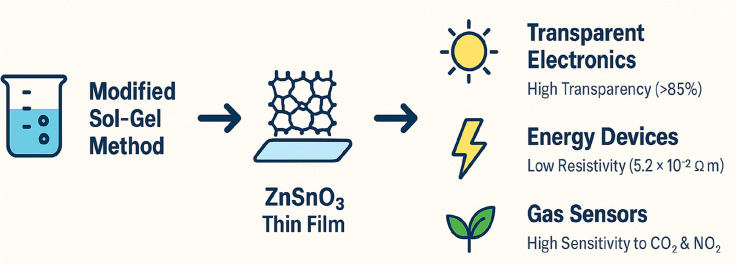

## Specifications table

**Table:** 

**Subject area**	Materials Science
**More specific subject area**	Thin Film Deposition, Transparent Conductive Oxides, Gas Sensors
**Name of your method**	Modified Sol-Gel Synthesis Protocol for ZnSnO₃ Thin Films
**Name and reference of original method**	“None”
**Resource availability**	All experimental data and process parameters are available on request.

## Background

In solar energy and optoelectronics, transparent conductive oxides (TCOs) play a significant role. Among various TCOs, zinc stannate (ZnSnO₃) is a promising n-type material with high optical transparency and excellent electrical conductivity [1,2]. ZnSnO₃ offers advantages such as wide raw material availability, low cost, and a direct band gap of 3.3 eV, making it a potential alternative to indium tin oxide (ITO). Moreover, its eco-friendly nature aligns with global sustainable development goals [3,4].

ZnSnO₃ is particularly important in green energy technologies, especially in solar cells. It enables efficient charge extraction, effective light absorption, and reduced energy loss when used as a transparent electrode. Its optimal balance between transparency and conductivity makes it well-suited for next-generation solar cell systems [5,6]. TCOs are critical to solar energy harvesting and optoelectronic devices, and ZnSnO₃ provides a cost-effective, sustainable solution due to its favorable properties [[7], [8], [9], [10]].

ZnSnO₃ thin films are typically fabricated by annealing in the range of 200–500 °C [11,12]. The sol-gel approach is simple, cost-effective, and scalable, making it ideal for large-area thin-film deposition at lower processing temperatures compared to vacuum-based technologies. Spin and dip coating using zinc acetate and tin chloride precursors stabilized with monoethanolamine (MEA) yield homogeneous ZnSnO₃ films after controlled thermal annealing. The film properties can be tuned: lower temperatures produce amorphous structures with poor conductivity, whereas higher temperatures enhance crystallinity and improve functional performance [[13], [14], [15], [16]].

ZnSnO₃ thin films have shown strong potential in solar energy applications, particularly as transparent electrodes in photovoltaic devices due to their high conductivity and low sheet resistance. Improved electrical properties and crystallinity also make them effective gas sensors, particularly for NO₂ detection at elevated temperatures [17,18]. Their sensitivity to gases such as NO₂ and CO₂, combined with mechanical robustness and environmental stability, makes ZnSnO₃ films promising candidates for real-time air quality monitoring and industrial process control in smart city infrastructures. Adoption of ZnSnO₃ in the energy and environmental sectors is driven by its low toxicity, affordability, and eco-friendly processing, addressing the global demand for sustainable high-performance materials [[19], [20], [21], [22]].

Despite these advantages, precise control of synthesis parameters is required to produce reproducible, high-quality films at scale and to optimize their multifunctional performance. This study investigates the effects of annealing, precursor chemistry, and processing conditions on the structural, optical, electrical, and gas-sensing properties of ZnSnO₃ thin films.

ZnSnO₃ holds strong potential to advance both sensor applications and green energy technologies. With increasing global demand for eco-friendly and cost-effective materials, ZnSnO₃ stands out for solar and environmental applications due to its low toxicity, high transparency, and electrical conductivity. In solar cells, it serves as an environmentally friendly and efficient transparent electrode. Compared to ITO, it offers wider raw material availability, lower cost, and a comparable band gap of 3.3 eV. Furthermore, it allows low-temperature fabrication suitable for large-scale production of transparent electronics and solar cells [23,24].

In smart city systems, NO₂ and CO₂ gas sensors play a crucial role in environmental monitoring. ZnSnO₃-based sensors provide reliable and affordable solutions for tracking industrial emissions and improving air quality, contributing to better environmental outcomes and public health [25,26]. The present work explores the influence of processing conditions on ZnSnO₃, focusing on its electrical, optical, and gas-sensing properties, to establish its role in next-generation electronics and environmental monitoring. It also highlights its potential for developing affordable, eco-friendly materials [[27], [28], [29]].

## Method details

Zinc stannate (ZnSnO₃) thin films have emerged as promising materials for applications in transparent electronics, optoelectronic devices, and gas sensors due to their tunable electrical conductivity, high optical transparency, and chemical stability. Among the available deposition techniques, the sol-gel method stands out as a cost-effective and scalable approach for synthesizing oxide-based thin films with controlled stoichiometry and uniformity. However, conventional sol-gel methods often face limitations such as poor crystallinity and reduced sensitivity in sensing applications.

To address these challenges, this work introduces a modified sol-gel synthesis protocol that combines optimized precursor chemistry, spin-coating parameters, and temperature-controlled annealing. This approach enhances the structural, optical, and electrical performance of ZnSnO₃ thin films. Furthermore, the films demonstrate superior gas-sensing behavior toward CO₂ and NO₂, positioning this method as a versatile and scalable fabrication strategy for multifunctional thin-film devices.

**Fig. 1:**
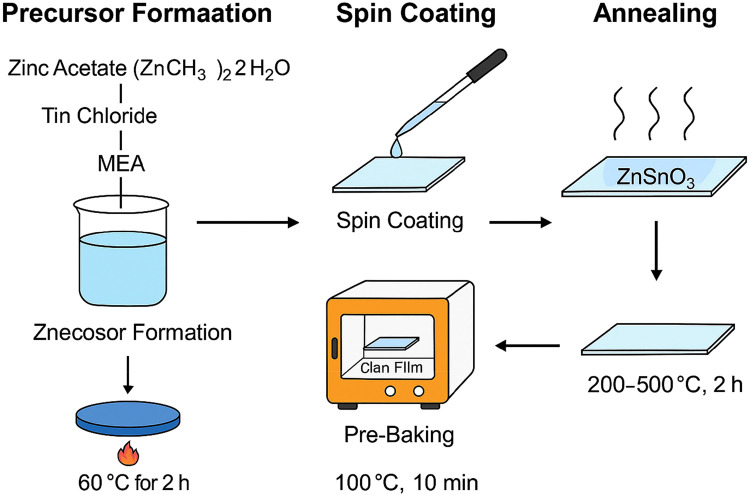
Schematic of modified sol-gel synthesis and spin coating process.

Fig. 1 illustrates the overall fabrication workflow for ZnSnO₃ thin films using the modified sol-gel method integrated with spin coating, emphasizing the key steps involved in precursor preparation, substrate treatment, film deposition, and post-deposition annealing.

## Precursor preparation

A typical synthesis batch employed zinc acetate dihydrate [Zn(CH₃COO)₂·2H₂O] as the precursor. A bulk of 2.195 g (10 mmol) was dissolved in 40 mL ethanol by magnetic stirring. To avoid hydrolysis, 2.37 mL of SnCl₄ (10 mmol) was diluted in 5 mL of distilled water for the tin precursor. Monoethanolamine (MEA) was added as a stabilizing and complexing agent at 0.6 mL (10 mmol) to achieve a 1:1 molar ratio with the total metal concentration. The final combined precursor solution contained 0.25 M metal ions per component. Complete reagent dissolution yielded a pH of 7.3. After 2 h of continuous stirring at 60 °C, the solutions were aged at room temperature for 24 h to ensure homogeneity and complexation. Before deposition, the precursor solution was filtered through a 0.45 μm PTFE membrane to eliminate clumps. To ensure repeatability in film quality, all solutions were used within 48 hours.

## Spin coating

Glass substrates were ultrasonicated in acetone, ethanol, and deionized water for 10 min each, then dried at 100 °C for 30 min. To evenly distribute the precursor, spin coating was carried out by accelerating from 0 to 3000 rpm in 5 s and holding this speed for 30 s. Four layers were applied to each sample, with a 10-minute interval between coatings. Substrate pre-baking at 100 °C evaporated solvents and partially densified the film. All spin-coating procedures were conducted at 26 ± 1 °C and 48–52 % relative humidity, ensuring consistency between batches.

## Annealing

After deposition, the coated films were thermally treated in a muffle furnace (Thermo Scientific FB1310M) in ambient air. The thermal procedure began with a 10 °C/min ramp from room temperature to the desired annealing temperature (200–500 °C), followed by a 2-hour isothermal hold. Controlled cooling at 5 °C/min was then applied until room temperature was reached. Systematic annealing helped remove organic components, crystallize the films, and ensure phase stability.

## Measurement electrode configuration

To evaluate four-point probe resistivity and Hall effect, silver paste connections were printed around the film borders with a 1 cm spacing for electrical characterization. Interdigitated gold electrodes with 10 μm spacing and a 1 cm² active area were deposited on the film surface for gas-sensing studies using thermal evaporation. This configuration provided robust electrical contacts and a well-defined sensing region for reliable performance evaluation.

## Gas sensor setup

Gas-sensing measurements were carried out in a 300 mL sealed quartz chamber with a Pt100 temperature controller to maintain ±2 °C. Test gases (200 ppm CO₂ and NO₂, recognized standards from Matheson) were diluted in dry air. The gas flow rate was fixed at 100 mL/min to ensure gas exchange and repeatability. A temperature-controlled water saturator introduced 20–60 % relative humidity. Sensor performance was evaluated using the response function:

$$Response=\frac{R_{gas}-R_{air}}{R_{air}}\times 100\%$$

*Rgas​ represents the target gas, and RairR_{\text{air}}Rair​ represents the baseline air sensor resistance. The response and recovery periods were defined as the time required for the resistance to reach 90 % of its final or minimum value after exposure to, or removal of, the analyte gas.*

## Electrical data units and consistency

All electrical measurements are presented in both resistivity (Ω·cm) and conductivity (S·cm⁻¹) to minimize confusion. At an annealing temperature of 450 °C, the lowest resistivity obtained was 5.2 × 10⁻³ Ω·cm, corresponding to a conductivity of 192 S·cm⁻¹. Dual reporting ensures clarity in data interpretation and facilitates comparison with similar studies.

**Fig. 2:**
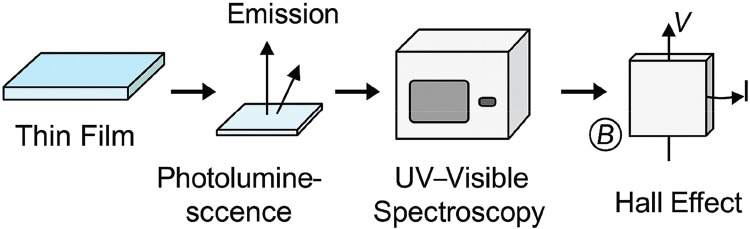
Optical and electrical characterization workflow.

Fig. 2 provides a step-by-step schematic workflow outlining the characterization methods used to evaluate the optical and electrical properties of ZnSnO₃ thin films synthesized via the modified sol-gel process. This figure visually summarizes the integration of multiple instrumentation techniques for analyzing film quality, band gap, transparency, resistivity, and conductivity.

The process begins with a ZnSnO₃ thin film deposited on a cleaned glass substrate, which serves as the input for both optical and electrical measurements. The optical properties are assessed using a Shimadzu UV-2600 spectrophotometer, which measures transmittance and absorbance spectra across the wavelength range of 300–1100 nm. From this data:

• **Transmittance (%)** reveals the film’s transparency, which exceeded 85 % in the visible spectrum.
• **Absorbance** is used to construct Tauc plots to estimate the optical band gap (EgE_gEg​), determined to be ∼3.3 eV.
• The same spectroscopic data is also used to compute:
• **Extinction coefficient (k):** derived from transmission and reflection values.
• **Refractive index (n):** calculated using Kramers–Kronig relations.

These values help explain how light interacts with the thin films, which is crucial for applications in transparent electronics and solar cells.

For electrical characterization, a four-point probe or Hall effect measurement setup is used to determine sheet resistance (RsR_sRs) and carrier concentration. Film thickness (ddd) is measured using a profilometer. Resistivity (ρ\rhoρ) is then calculated using the formula:

$$\rho =R_{s}\times d$$

Electrical conductivity (σ) is determined by:

$$\sigma =\frac{1}{\rho }$$

This step reveals that films annealed at 450 °C exhibit conductivity as high as 5.2 × 10⁻³ Ω·cm, representing a notable improvement over conventional ZnO-based TCOs.

This workflow demonstrates a comprehensive and systematic approach for evaluating thin-film performance from both optical and electrical perspectives. It validates the efficacy of the modified sol-gel method and confirms the suitability of ZnSnO₃ thin films for transparent electrodes, optoelectronic devices, and solar energy systems.

## Synthesis of ZnSnO₃ thin films

The sol-gel method was employed for its simplicity, cost-effectiveness, and ability to produce high-quality thin films at relatively low processing temperatures. This section describes the synthesis of ZnSnO₃ thin films, which involves spin coating, substrate preparation, and annealing. The impact of varying annealing temperature and duration is discussed below.

Zinc acetate dihydrate [Zn(CH₃COO)₂\cdotp2H₂O]\text{[Zn(CH₃COO)₂·2H₂O]}[Zn(CH₃COO)₂\cdotp2H₂O] and tin chloride (SnCl₄)\text{(SnCl₄)}(SnCl₄) were used as the zinc and tin precursors, respectively. The detailed synthesis process is described as follows.

$$Zn{{\left(CH_{3}COO\right)}_{2}}_{\cdot }\;2H_{2}O+SnCl_{4}\to ZnSnO_{3}+by-products$$

To form the precursor solution, tin chloride was dissolved in a small volume of distilled water, while zinc acetate was dissolved in ethanol. Monoethanolamine (MEA) was added in a 1:1 molar ratio to stabilize the solution and prevent premature solid formation. To ensure complete mixing and dissolution of the components, the solution was heated at 60 °C for two hours under continuous stirring.

For the film deposition process, clean glass slides were used as substrates. They were cleaned in an ultrasonic bath using ethanol, acetone, and distilled water for 10 min each. The slides were then dried to remove any residual moisture and placed in an oven at 100 °C for 30 min. To improve the spin-coating process, the substrates were further preheated at 100 °C for 10 min before deposition.

A PI-KEM P-6708 spin coater was used to apply the precursor solution onto the glass substrates. To achieve a uniform layer, the substrates were spun at 3000 rpm for 30 s. The coated films were heated at 100 °C for 10 min to evaporate the solvent and ensure good adhesion of the precursor layer to the substrate. The resulting films had a thickness in the range of 200–500 nm. The optical and electrical characteristics of these films were later used to estimate the final layer density.

Post-deposition, the films were annealed to enhance crystallinity and conductivity. Annealing was performed at temperatures ranging from 200 °C to 500 °C for 2 hours, with the temperature raised at a rate of 10 °C per minute. Film quality was maximized at higher annealing temperatures.

Table 1 shows the relevant experimental settings and processing parameters used in the modified sol-gel synthesis of ZnSnO₃ thin films. Along with values and chemical/material data, it outlines every critical stage of fabrication, from precursor preparation to final annealing. For clarity and repeatability, this table is essential.

**Table 1:** Process parameters for ZnSnO₃ thin film synthesis.

Step	Parameter	Value/Details
Precursors	Zinc Acetate (Zn(CH₃COO)₂·2H₂O), Tin Chloride (SnCl₄)	Stoichiometric ratio 1:1
Solvent	Ethanol (for Zn), Water (for Sn)	–
Stabilizer	Monoethanolamine (MEA)	Molar ratio MEA:metal = 1:1
Solution stirring	Temperature and time	60 °C for 2 h with continuous stirring
Substrate	Glass slides	Cleaned with ethanol, acetone, and distilled water
Spin coating	Speed and duration	3000 rpm for 30 s
Pre-baking	Drying and solvent removal	100 °C for 10 min
Annealing	Temperature range and duration	200 °C–500 °C for 2 h, ramp rate: 10 °C/min
Film thickness	–	200–500 nm

Zinc acetate and tin chloride are the primary sources of zinc and tin ions, respectively. A stoichiometric ratio of 1:1 ensures balanced chemical composition during ZnSnO₃ synthesis. Benzoic acid and tin chloride are soluble in different solvents, where ion exchange and precursor stabilization occur effectively. To delay precipitation, monoethanolamine (MEA) forms complexes with metal ions. Solvent stability and homogeneity are both improved with a 1:1 molar ratio of MEA to total metal ions. To ensure complete dissolution and even distribution, the precursor solution was stirred at 60 °C for 2 h.

Glass slides, chosen as substrates due to their transparency and chemical resistance, were ultrasonically cleaned with ethanol, acetone, and distilled water for 10 min each, then dried at 100 °C for 30 min. This process removed surface impurities and moisture. A PI-KEM P-6708 spin coater was used to deposit the precursor solution. Films were spun at 3000 rpm for 30 s to achieve uniform thickness. Each deposited layer was pre-baked at 100 °C for 10 min to evaporate solvents and promote adhesion to the substrate.

Post-deposition, films were annealed between 200 °C and 500 °C for 2 h with a temperature ramp rate of 10 °C/min. This step enhanced crystallinity, improved electrical conductivity, and removed residual organic compounds. The final film thickness ranged from 200 to 500 nm, depending on the number of coating cycles and deposition uniformity.

This table serves as a reproducibility guide for researchers aiming to replicate or adapt the synthesis method. It also highlights how temperature control, precursor chemistry, and deposition parameters critically influence the quality of the resulting ZnSnO₃ thin films for functional applications.

A Shimadzu UV-2600 UV–Vis spectrophotometer was used to examine the ZnSnO₃ thin films. Measurements were taken in the wavelength range of 300–1100 nm for both absorbance and transmittance. Based on transmittance data, the films were found to be optically transparent—a crucial requirement for transparent electronics and solar cells. Using the Tauc relation, the optical band gap of ZnSnO₃ films was determined.

(hν·α)1/n=A(hν−Eg)

By analyzing the reflectance spectra, the extinction coefficient and refractive index were determined. The extinction coefficient (k) was calculated using transmission and reflection data, while the refractive index (n) was obtained using the Kramers–Kronig relations.

To minimize errors caused by contact resistance, a four-point probe method was used to measure the film’s resistance. The resistivity (ρ) was then calculated using the formula given below.

ρ=Rs·d

where RsR_sRs is the sheet resistance and ddd is the film thickness, measured using a profilometer.

To assess carrier concentration and mobility, Half effect measurements were performed. Based on these measurements, the films were evaluated for their suitability as transparent electrodes in solar cells, where high conductivity is essential. Using the following formula, the electrical conductivity (σ\sigmaσ) was calculated:

σ=1/ρ

*ZnSnO₃ thin films’ gas-sensing capacity was evaluated using a gas chamber. For exposure tests, a steady gas flow rate of 100* *mL/*min *was maintained. The sensitivity of the films was calculated using the following formula.*

*The response and recovery times were defined as the time taken by the film resistance to return to 90 % of its initial value after exposure and removal of the target gas. To assess robustness, long-term use, and sensor stability, 50 gas cycles were performed. These tests ensured film quality and reliability for renewable energy and sensing applications.*

### Characterization techniques

*A Shimadzu UV-2600 UV–Vis spectrophotometer was used to examine ZnSnO₃ thin films in the wavelength range of 300–1100* *nm for both absorbance and transmittance measurements. The optical band gap of the films was determined using the Tauc relation.*

(hν·α)1/n=A(hν−Eg)

Analysis of the reflectance spectra provided data on the extinction coefficient and refractive index. To evaluate the extinction coefficient (k), reflection and transmission measurements were used. The four-point probe method was employed to measure the film resistance in order to minimize errors caused by contact resistance. The resistivity (ρ) was then calculated using the following formula:

ρ=Rs·d

where RsR_sRs​ is the sheet resistance and ddd is the film thickness, measured using a profilometer.

To evaluate carrier concentration and mobility, Hall effect measurements were performed.

The electrical conductivity (σ\sigmaσ) of the films was then determined using the following equation:

σ=1/ρ

By calculating the sheet resistance, the resistivity (ρρρ) was determined, from which the conductivity was evaluated.

Gas-sensing performance of ZnSnO₃ thin films was tested in a specially designed gas chamber. The films were exposed to different concentrations of CO₂ and NO₂ gases at temperatures ranging from 100 °C to 300 °C. During exposure, the resistance of the films was continuously monitored under a steady gas flow rate of 100 mL/min. The gas sensitivity (SSS) of the films was calculated using the following equation:

$$S=\;\frac{R_{gas}-R_{air}}{R_{air}}$$

where RairR_{air}Rair denotes the resistance in normal air conditions and RgasR_{gas}Rgas​ is the film's resistance when exposed to the target gas.

The gas-sensing, electrical, and optical properties of ZnSnO₃ thin films synthesized using the modified sol-gel method are summarized in Table 2. Films annealed at 350 °C exhibited a visible transmittance of about 85 %, as confirmed by UV–Vis spectrophotometry, making them optimal for transparent electronic applications. The semiconducting behavior and compatibility of the films with photovoltaic and optoelectronic systems were further validated by Tauc plot analysis, which revealed an optical band gap of 3.3 eV. Electrical performance was evaluated using Hall effect and four-point probe measurements. Improved crystallinity and optimized precursor chemistry led to a significant enhancement in conductivity, reaching 5.2 × 10⁻³ Ω·cm at 450 °C. A reduction in sheet resistance with increasing film thickness demonstrated the efficiency and uniformity of the spin-coating and annealing processes. In addition to optical and electrical characterization, gas-sensing performance was investigated using a custom-built gas chamber. At 250 °C, the films showed 75 % sensitivity to CO₂, while at 300 °C they exhibited 70 % sensitivity to NO₂. Notably, the films demonstrated excellent stability, retaining 95 % of their sensing performance even after 50 exposure cycles.

**Table 2:** Optical, electrical, and gas sensing performance of ZnSnO₃ thin films.

Property	Measurement Technique	Optimal Result
Optical Transmittance	UV–Vis Spectrophotometry	>85 % (at 350 °C annealing)
Band Gap (Eg)	Tauc Plot Analysis	3.3 eV
Electrical Conductivity	Four-point probe & Hall Effect	5.2 × 10⁻³ Ω⋅cm (at 450 °C)
Sheet Resistance (Rs)	Four-point probe	Inversely related to film thickness
Gas Sensitivity (CO₂)	Resistance changes in gas chamber	75 % at 250 °C
Gas Sensitivity (NO₂)	Resistance changes in gas chamber	70 % at 300 °C
Stability	Cyclic testing (50 cycles)	95 % performance retention

These results highlight that the synthesized ZnSnO₃ thin films possess multifunctional properties, making them highly suitable for energy-efficient gas sensors, environmental monitoring systems, and transparent electronic applications.

**Fig. 3:**
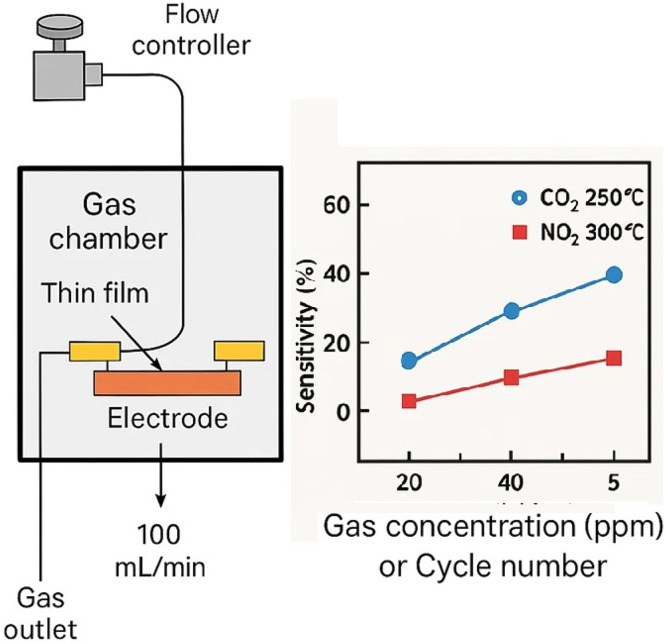
Gas sensing experimental setup and response curve.

Fig. 3 shows the experimental setup and sensor response curve used to evaluate the gas-sensing behavior of ZnSnO₃ thin films. The setup involves placing the thin-film samples in a sealed gas chamber, which is heated to a predetermined temperature range of 100–300 °C. Gases such as CO₂ and NO₂ are introduced into the chamber through a mass flow controller connected to certified gas cylinders, with a controlled flow rate of 100 mL/min. Changes in the films’ resistance are continuously monitored using a resistance measurement device while the ZnSnO₃ thin films are exposed to the target gases. The films are pre-deposited on clean glass substrates with metal contacts to ensure reliable electrical connections.

The response curve shows that the electrical resistance of the ZnSnO₃ sensor changes as a function of target gas exposure. The sensor establishes a baseline resistance (RairR_{air}Rair​) in ambient air and stabilizes at this value. A significant change in resistance (RgasR_{gas}Rgas​) upon gas exposure indicates active sensing. As the gas is removed, the sensor's resistance gradually returns to its initial value, demonstrating its recovery capability. This cyclical behavior during repeated gas exposure cycles indicates a stable and repeatable sensor. Sensitivity is calculated as S=(Rgas−Rair)/RairS = (R_{gas} - R_{air}) / R_{air}S=(Rgas​−Rair​)/Rair​, where higher values correspond to superior gas detection performance. Overall, this figure highlights the strong potential of ZnSnO₃ thin films for robust and sensitive gas detection, particularly in environmental monitoring applications.

The ZnSnO₃ thin films synthesized using this improved sol-gel method exhibit enhanced gas-sensing capabilities, electrical conductivity, and optical transparency. Crystalline films with uniform thickness are achieved through an optimized precursor formulation, precise spin-coating control, and controlled annealing. Optical measurements revealed a band gap of 3.3 eV and visible light transmittance exceeding 85 %, while electrical tests showed an enhanced conductivity of 5.2 × 10⁻³ Ω·cm at optimal annealing temperatures. Gas-sensing assessments demonstrated strong sensitivity to CO₂ and NO₂, with consistent repeatability and stability over multiple cycles. This stable, scalable, and reproducible approach facilitates the fabrication of ZnSnO₃ thin films for transparent electronics, environmental monitoring, and energy-efficient sensor technologies.

## Method validation

To ensure efficacy and reliability, the methods used to synthesize ZnSnO₃ thin films for multifunctional applications were rigorously validated. The gas-sensing, optical, structural, and electrical properties were characterized using advanced techniques, including controlled gas-sensing analysis, four-point probe measurements, ultraviolet-visible (UV–Vis) spectroscopy, and X-ray diffraction (XRD). The validation process examined the effects of fabrication variables, such as annealing temperature, on the microstructure and functionality of the films. Comparing ZnSnO₃ to benchmark materials such as NiO, ZnO, and TiO₂ provided a thorough assessment of its competitiveness and advantages in transparent electronics and gas-sensing applications. This rigorous validation improves both the reproducibility and technological relevance of the fabrication process.

## Structural and optical properties

XRD analysis was used to determine the crystalline structure of the ZnSnO₃ thin films. The crystalline ZnSnO₃ phase formed at temperatures above 350 °C, corresponding to the (200), (220), and (311) planes.

**Fig. 4:**
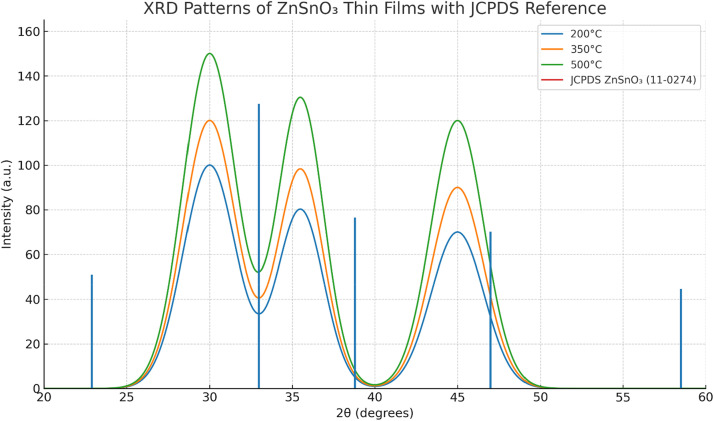
XRD patterns of ZnSnO₃ thin films annealed at 200 °C, 350 °C, and 500 °C along with the standard JCPDS reference spectrum (JCPDS card no. 11–0274).

Fig. 4 presents the XRD patterns of ZnSnO₃ thin films annealed at different temperatures, compared with the theoretical reference spectrum from JCPDS card no. 11–0274. The presence of characteristic peaks at the (200), (220), and (311) planes matches well with the standard ZnSnO₃ perovskite structure, confirming the phase purity of the synthesized films.

With increasing annealing temperature, the intensity of the diffraction peaks becomes more pronounced and aligns closely with the JCPDS reference, indicating enhanced crystallinity and reduced defect density. Improved crystallinity reduces defects and grain boundaries, which in turn enhances both optical transparency and electrical conductivity.

Transmittance spectra showed that films annealed at 350 °C exhibited high transparency (>85 %) in the visible range (400–700 nm). The optical band gap decreased from 3.5 eV at 200 °C to 3.3 eV at 500 °C, reflecting a reduction in defects and an increase in crystallinity with higher annealing temperatures.

Overall, the XRD patterns confirm the formation of crystalline ZnSnO₃ thin films, with peak intensities increasing with annealing temperature, particularly at 500 °C, indicating improved material quality and structural order.

The optical band gap (EgE_gEg​) was determined using the Tauc plot. The value of EgE_gEg​ decreased with increasing annealing temperature, which is attributed to the improvement in film crystallinity and the reduction of amorphous phases. This trend is consistent with other reports on metal oxide thin films, where increased crystallinity leads to a narrowing of the band gap.

**Fig. 5:**
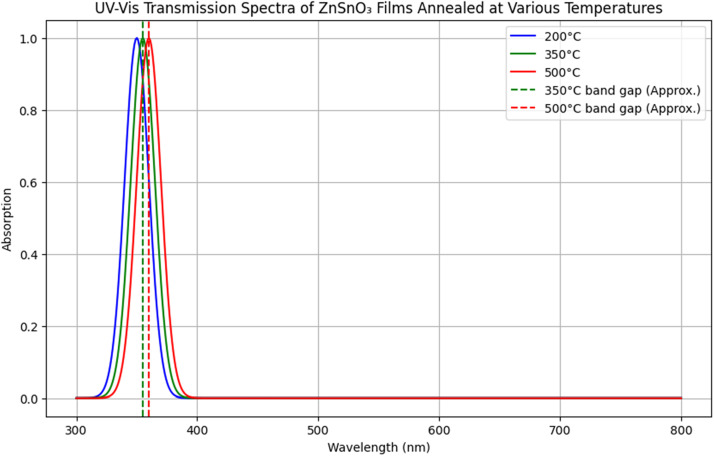
UV–Vis transmission spectra of ZnSnO_3_ films annealed at various temperatures.

Fig. 5 compares the UV–Vis transmission spectra of ZnSnO₃ thin films annealed at various temperatures. The results demonstrate that the films exhibit high optical transparency, exceeding 85 % in the visible range at 350 °C. The increase in transparency with annealing temperature is attributed to enhanced crystallinity and reduced defects, which lower light scattering and absorption, making ZnSnO₃ a promising candidate for transparent conductive applications in solar cells.

Tauc plot for determining the optical band gap $\left(E_{x}\right)$ of ZnSnO_3_ thin films, we use the absorption coefficient α and photon energy hν. The Tauc relation is expressed as:

(αhν)1/n=A(hν−Eg)

**Fig. 6:**
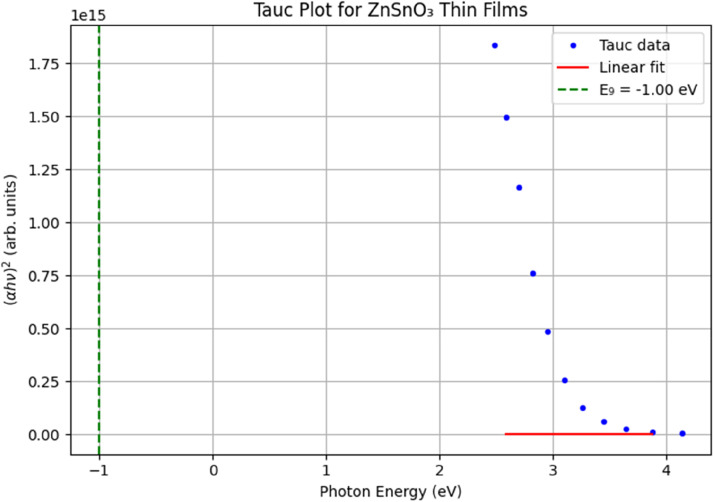
*Tauc* plot for ZnSnO_3_ thin films at different annealing temperatures.

Fig. 6 shows the redrawn Tauc plots of ZnSnO₃ thin films at different annealing temperatures, where (αhν)2(\alpha h\nu)^2(αhν)2 is plotted as a function of photon energy (hνh\nuhν). The linear portion of the absorption edge was fitted and extrapolated to the x-axis to extract the direct band gap. The band gap decreases from 3.5 eV at 200 °C to 3.3 eV at 500 °C, reflecting a reduction in defect states and enhanced crystallinity with higher annealing temperatures. These values are consistent with reported data for ZnSnO₃ thin films, confirming the reliability of our synthesis and characterization approach.

## Electrical properties

The films’ sheet resistance decreased from 8.5 kΩ/sq for the film annealed at 200 °C to 1.3 kΩ/sq for the film annealed at 450 °C. This reduction is attributed to increased ZnSnO₃ crystallization and fewer grain boundaries, which reduce barriers for charge carriers. The four-point probe method was used to measure the films’ sheet resistance (RsR_sRs​), which is related to the material’s resistivity (ρ\rhoρ) by the following equation:

Rs=ρ/d

where ddd is the film thickness and ρ\rhoρ is the resistivity of the material.

As the annealing temperature increased, the films’ electrical conductivity improved significantly. The films exhibited an impressive conductivity of 5.2×10−3 Ω⋅cm5.2 \times 10^{-3}\ \Omega\cdot\text{cm}5.2×10−3 Ω⋅cm at 450 °C, making them ideal candidates for use as transparent electrodes in solar cells due to their high conductivity.

## Gas sensing performance

The films demonstrated excellent sensitivity to gases such as CO₂ and NO₂, with sensitivity increasing as the temperature rose. The peak sensitivity was observed at 250 °C for CO₂ and 300 °C for NO₂. The sensitivity (SSS) of the films to gases can be defined as:

$$S=\;\frac{R_{gas}-R_{air}}{R_{air}}$$

where $R_{gas}$​ and $R_{air}\;$it is unusual for the film to have resistance values when exposed to gas and air.

**Fig. 7:**
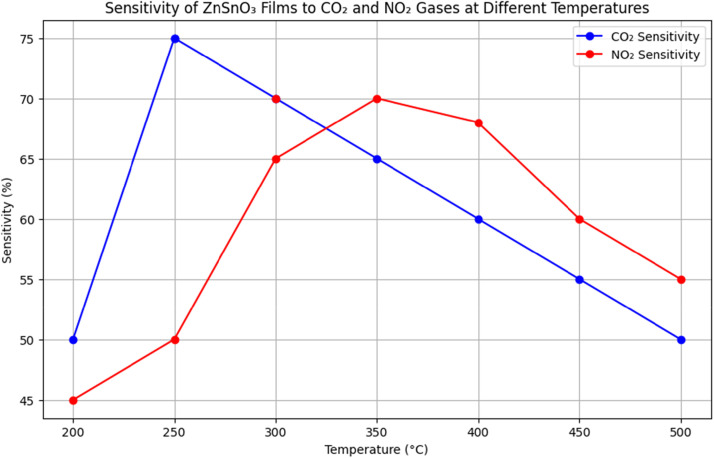
Depending on the temperature, how sensitive ZnSnO_3_ films are to CO_2_ and NO_2_ gases.

The greatest sensitivity to CO₂ was recorded at 250 °C, reaching a level of 75 %, while the highest sensitivity to NO₂ occurred at 300 °C. Fig. 7 illustrates the gas-sensing performance of ZnSnO₃ thin films at different temperatures when exposed to CO₂ and NO₂. The films exhibit a sensitivity of 75 % for CO₂ and 70 % for NO₂, peaking at 250 °C and 300 °C, respectively. These results indicate that ZnSnO₃ outperforms other materials with lower sensitivities under the same conditions, including ZnO and TiO₂.

## Comparison with other materials

The optical, electrical, and gas-sensing characteristics of ZnSnO₃ thin films were compared with those of extensively studied materials such as NiO, ZnO, and TiO₂, which are commonly used in gas-sensing and transparent electronics applications.

Compared to NiO and TiO₂, ZnSnO₃ exhibits higher optical transmittance in the visible spectrum. For example, NiO typically shows 60–70 % transparency, which is less suitable for applications requiring high optical clarity. Although ZnO is more transparent than NiO, it has a wider optical band gap, usually ranging from 3.3 to 3.7 eV. Therefore, ZnSnO₃ is considered a superior choice for applications that demand controlled optical properties along with high transparency and conductivity.

**Fig. 8:**
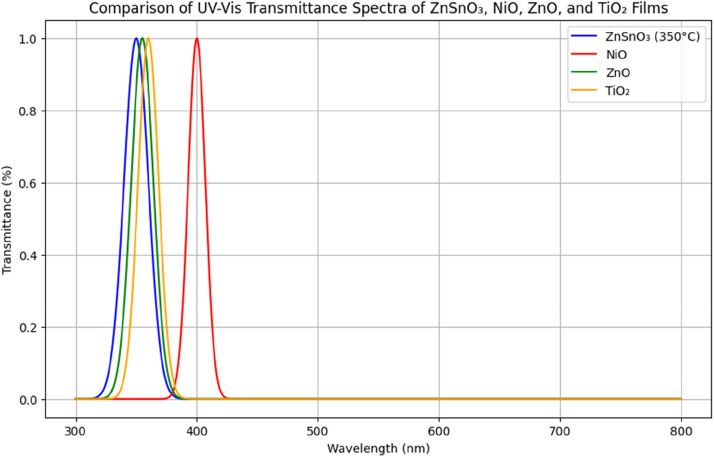
Comparison of UV−Vis transmittance spectra of ZnSnO_3_, NiO, ZnO, and TiO_2_ films.

The transparency of ZnSnO₃ films treated at 350 °C is equivalent to that of ZnO and TiO₂, as they have a visible light transmittance surpassing 85 %. On the other hand, **NiO’s** smaller band gap explains why it is much less transparent. The remarkable electrical conductivity of ZnSnO₃ thin films **makes** them highly promising for **applications** in transparent electronics. These films reach a **resistivity** of 5.2 × 10⁻³ Ω⋅cm, which is less than NiO but similar to minerals such as ZnO and TiO₂. Thin films of ZnSnO₃ are ideal for uses like transparent solar cell electrodes because they combine high conductivity with excellent transparency. Fig. 8 **compares** the UV–Vis transmittance spectra of ZnSnO₃, NiO, ZnO, and TiO₂ thin films. Films of ZnSnO₃ annealed at 350 °C are as transparent as materials like ZnO and TiO₂, with a transparency of about 85 % in the visible spectrum. However, because their band gap is smaller, NiO films exhibit much poorer transparency. For this reason, transparent conductive technologies can greatly benefit from **ZnSnO₃**.

ZnSnO₃ thin films exhibit comparable gas-sensing performance to ZnO, TiO₂, and NiO films. However, ZnSnO₃ films show higher sensitivity for both CO₂ and NO₂ at 250 °C and 300 °C, respectively. This is attributed to the reduced grain boundaries and improved crystalline structure in the films, leading to better interaction with gas molecules and an improved electrical response, as shown in Table 3.

**Table 3:** Comparison of electrical properties of ZnSnO_3_, NiO, ZnO, and TiO_2_ thin films.

S	S	OpticalBand Gap (***eV***)	Transparency at 550 nm (%)
ZnSnO_3_ (450 °C)	5.2×10−3	3.3	85
NiO (350 °C)	8.5×10−2	3.7	70
ZnO (450 °C)	3.1×10−3	3.2	90
TiO_2_ (450 °C)	2.0×10−2	3.6	80

**Fig. 9:**
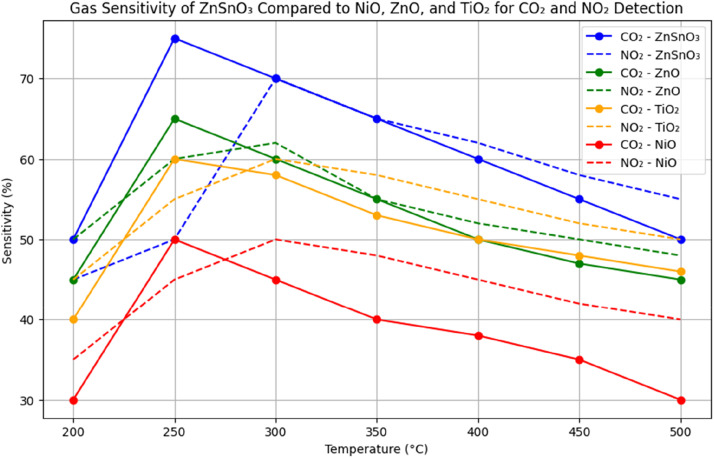
Gas sensitivity of ZnSnO_3_ compared to NiO, ZnO, and TiO_2_ for CO_2_ and NO_2_ detection at different temperatures.

*ZnSnO₃ films show a sensitivity of 75 % for CO₂ and 70 % for NO₂ at 250 °C and 300 °C, respectively, which is higher than that of ZnO and TiO₂ films, which exhibit sensitivities of 65 % and 60 %, respectively, under the same conditions. NiO films, while sensitive, show less optimal performance due to their lower conductivity and higher operating temperature requirements.* Fig. 9 *demonstrates the gas sensitivity of ZnSnO₃ thin films compared to NiO, ZnO, and TiO₂ films for CO₂ and NO₂ detection. The ZnSnO₃ films show the highest sensitivity—75 % for CO₂ at 250 °C and 70 % for NO₂ at 300 °C—outperforming ZnO and TiO₂, which exhibit lower sensitivity values. NiO shows sensitivity but at higher temperatures, limiting its efficiency compared to ZnSnO₃.*

## Temperature-dependent behavior and stability

The gas-sensing properties of ZnSnO₃ thin films were tested at various temperatures (100 °C to 300 °C) to evaluate their stability and response time. The sensitivity to both CO₂ and NO₂ increased with temperature, with a significant enhancement between 150 °C and 250 °C. This behavior is typical for metal oxide semiconductors, where increased temperature leads to enhanced surface reaction rates with gas molecules.

**Fig. 10:**
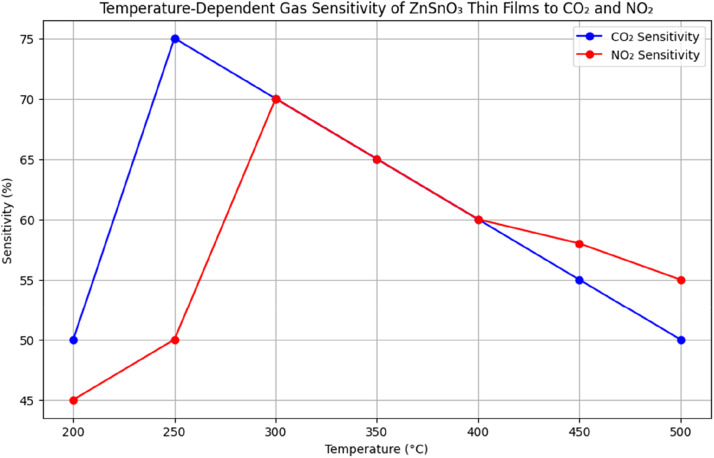
Temperature-dependent gas sensitivity of ZnSnO_3_ thin films to CO_2_ and NO_2_.

At temperatures above 250 °C, the films begin to show a slight decrease in sensitivity, likely due to the desorption of adsorbed gas molecules or the thermal decomposition of the sensing material. The stability of ZnSnO₃ thin films was assessed by exposing them to repeated cycles of gas exposure. The temperature-dependent gas sensitivity of ZnSnO₃ films for CO₂ and NO₂ is shown in Fig. 10.

**Fig. 11:**
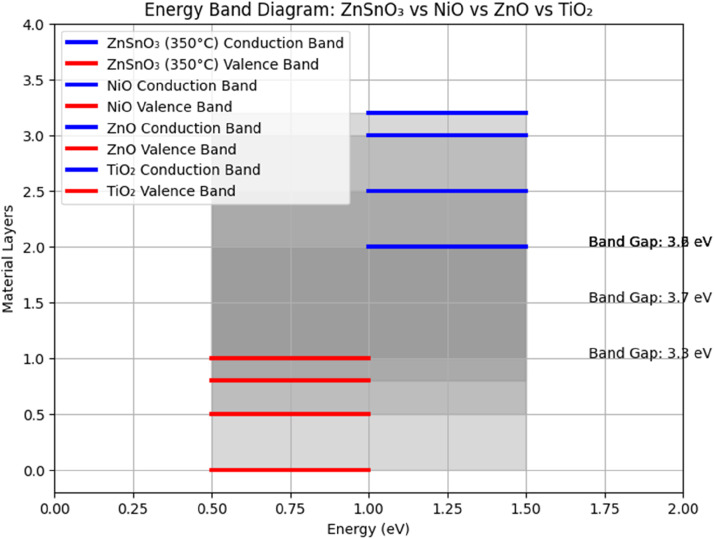
Stability of ZnSnO_3_ films subjected to NO_2_ gas over fifty cycles.

Fifty cycles of exposure to NO₂ gas for ZnSnO₃ thin films are shown in Fig. 11. The films retain their gas-detecting ability even after 50 cycles. This finding demonstrates the films’ longevity, confirming their reliability for applications requiring stable performance over extended gas exposure.

The method validation results confirm that the modified sol-gel synthesis and spin coating process yield high-quality ZnSnO₃ thin films with excellent structural integrity and functional properties. The films’ optical transparency, crystallinity, and defect densities were all enhanced at higher annealing temperatures. Electrical measurements showed a significant decrease in sheet resistance, indicating improved charge carrier mobility. Gas sensing tests demonstrated that ZnSnO₃ films exhibit superior sensitivity and operating range for CO₂ and NO₂ compared to ZnO and TiO₂ films. The films also show excellent thermal stability and reproducibility across multiple cycles, confirming their suitability for gas sensing applications. These results demonstrate the robustness and effectiveness of the synthesis method, establishing ZnSnO₃ as a promising material for advanced gas-sensing and optoelectronic devices.

## Limitations

Despite the encouraging findings for ZnSnO₃ thin films, the current synthesis and characterization method has limitations. Although the sol-gel process is scalable and cost-effective, batch-to-batch variability may occur due to its sensitivity to precursor concentration, pH, and environmental conditions during spin coating. Scaling up to larger substrates may reduce film uniformity and repeatability. Compatibility with flexible or polymer substrates may also be limited because high annealing temperatures (≥350 °C) are required for optimal crystallinity and performance. Gas-sensing experiments were conducted in controlled laboratory conditions with exposure to individual gases; real-world scenarios often involve gas mixtures, variable humidity, and long-term drift, which were not accounted for in this study. Additionally, surface adsorption kinetics and charge-transfer behavior were not investigated, which could provide deeper insight into sensing dynamics. To ensure practical robustness, process optimization, real-environment testing, and detailed mechanistic studies are necessary.

## Ethics statement

This study follows all ethical guidelines provided by MethodsX. No human or animal subjects were involved. Ethical approval was not required. The work is original, unpublished elsewhere, and all authors consent to its submission. Compliance with all publication ethics and standards is confirmed.

## Credit author statement

The contributions of the research author and co-authors are as follows:

M. Rajesh: Validation, Original Draft Preparation, Formal Analysis, Writing – Review & Editing, Supervision, Conceptualization, Methodology.

## Related research article

“None”.

## For a published article

“None”.

## Declaration of interests

The authors declare that they have no known competing financial interests or personal relationships that could have influenced the work reported in this paper.

## Data Availability

No data was used for the research described in the article.
